# Decreasing loco-regional recurrence for oral cavity cancer with total Mohs margins technique

**DOI:** 10.1186/s40463-016-0176-9

**Published:** 2016-12-01

**Authors:** Mathieu Bergeron, Pierre Gauthier, Nathalie Audet

**Affiliations:** 1 Department of Ophthalmology and Otolaryngology – Head & Neck Surgery, Faculty of Medicine, Laval University, 1050, avenue de la Médecine, Pavillon Ferdinand-Vandry, bureau 4889, Québec, Québec G1V 0A6 Canada; 2 Department of Otolaryngology– Head & Neck Surgery, Centre Hospitalier Régional de Launaudière, 1000 Boulevard Ste-Anne, St-Charles-Borromée, Québec J6E 6J2 Canada; 3 Department of Otolaryngology – Head & Neck Surgery, CHU de Québec-Université Laval Hôpital de l’Enfant-Jésus, 2705, 1401 18e rue, Québec, Québec GIJ 1Z4 Canada

**Keywords:** Mohs, Margins, Oral cavity, Cancer, Recurrence, Frozen margins, Tongue, Revision, Pathology, Squamous cell carcinoma

## Abstract

**Background:**

The conventional technique for cancer resection margin analysis studies only 0.1% of the surgical margins. Complete frozen section margins - also known as Mohs margins – allows for analysis of 100% of the surgical margins.

**Methods:**

The objective of our study is to compare oral cavity cancer loco-regional recurrence rates when treated by total frozen sections technique (Total Mohs margins) versus conventional margins. We conducted a multicenter retrospective cohort chart review. Loco-regional oral cancer recurrence rates were compared between patients treated with total Mohs margins (2007–2013) and patients treated with conventional margins techniques (2002–2007).

**Results:**

After applying inclusion criteria, a total of 60 patients treated by total Mohs margins and 57 patients with conventional margins were identified. Patients had similar baseline cancer stages, pathological types, past head and neck cancers and comorbidities (all *p* > 0.05). One-year recurrence rate was lower (10.0% vs 21.1%, *p* = 0.019) in favor of Mohs total margins and stayed significantly lower at 5 years of follow-up. When adjusted for T grade with N0 disease, Mohs technique was still beneficial in loco-regional recurrence for Tis-T4N0 up to 2 years (10.5% vs 25.7%, z-score 1.849, *p* = 0.032). The Number Needed to Treat at 2 years of follow-up for this subgroup of patients (Tis-T4N0) is 6.6. Margins had to be retaken more often intra-operatively in Mohs technique (68.3% vs 12.3%, *p* < 0.0001), mainly for positive deep margins (48.6% of all margins, *p* = 0.028). Duration of surgery was not increased with Mohs vs conventional technique (380 min vs 475 min respectively, *p* = 0.025).

**Conclusions:**

Mohs total margins may result in a significant reduction in cancer recurrence rate at 5 years compare to conventional surgery. Moreover, duration of surgery was not increased when using Mohs technique when judiciously performed.

**Electronic supplementary material:**

The online version of this article (doi:10.1186/s40463-016-0176-9) contains supplementary material, which is available to authorized users.

## Background

**Fig. 1:**
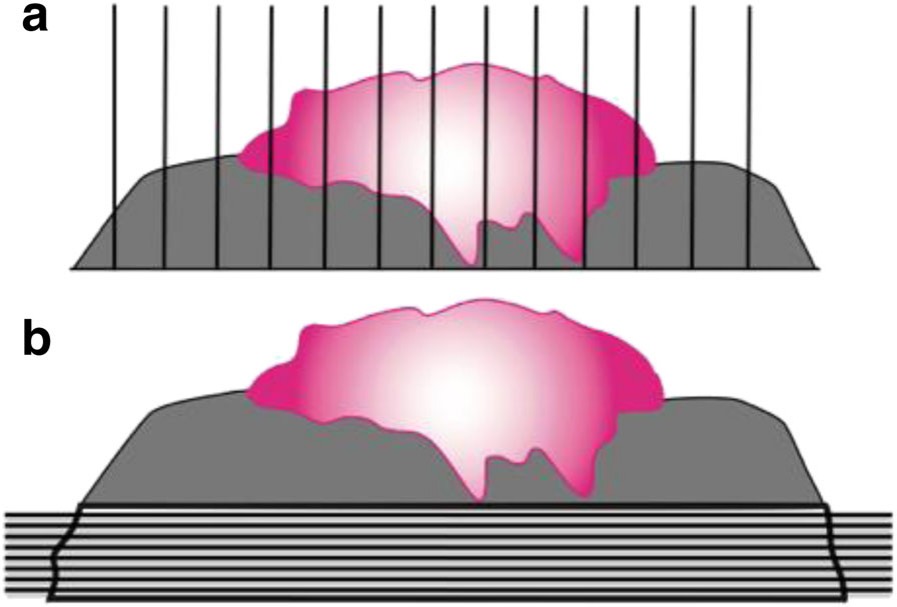
Total Mohs vs Conventional margins. **a** demonstrates conventional margins (vertically taken), which can miss positive margin. **b** demonstrates Mohs technique in the margin specimen which will analyse 100% of the margins (horizontal slices)

According to Davidson, the conventional technique for cancer resection margin analysis studies only 0.1% of the surgical margins [1, 2]. The presence of a positive margin is a well-known risk factor for cancer recurrence. It is therefore paradoxical that we analyze less than one percent of them [2]. When peri-operative margins are positive, many surgeons send further fragments of tissue from the resected site for analysis. Pathologists receive these small and un-oriented fragments and frequently take only samples from them [3]. However, use of complete frozen section margins - also known as Mohs margins – allows for analysis of 100% of the margins. (Fig. 1) [2, 4] Globally, Mohs surgery allows tumor excision and microscopic evaluation of the entire peripheral and deep margins. Residual tumor identified on histologic examination is marked on a pictorial map to guide the removal in subsequent stages until negative margins are achieved. Mohs surgery is already commonly used in non-melanoma skin cancer removal, for which it has been shown to decrease local recurrence [5]. Furthermore, it is useful to preserve healthy tissue in critical zones, which is of particular importance in head and neck oncology [6]. Indeed, this technique has been adapted for mucosal lesion in the head and neck region in the 80s, with promising results [1, 4].

**Fig. 2:**
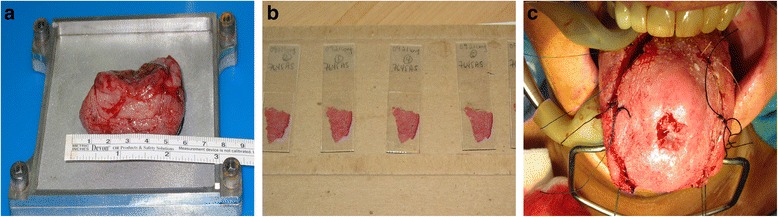
Steps from molds conception to case series. **a** (step 1): mold conception. Molds adapted for H&N reality. Flatten the specimen with more efficiency. **b** (step 2): animal experience. Good quality with sharp dissection. Isopentane and cryostat offer good results. **c** (step 3): case series. Clear margins in oriented specimen. No short-term recurrence (*n* =12)

Animal data supports performing Mohs margins for oral cavity cancer. Given the potential benefits in humans, our group has performed routine Mohs margins for oral cavity cancer since 2007 (Fig. 2) [7, 8].

However, few studies have evaluated the usefulness of frozen sections for positive margins in oral cavity cancer [9]. To our knowledge, no studies have specifically looked at the usefulness of complete frozen margin resection in decreasing oral cavity cancer recurrence rates. In cases of non-melanoma skin cancer, Mohs technique has been shown to increase clear margins and decrease local recurrence rates [10]. Given these preliminary data, we hypothesized that Mohs margins could lead to a reduced oral cavity loco-regional recurrence rate. Therefore, the aim of this study was to evaluate oral cavity cancer loco-regional recurrence rates when using Mohs margins compared to conventional technique for primary squamous cell carcinoma (SCC) of the oral cavity.

## Methods

### Total Mohs margins

**Fig. 3:**
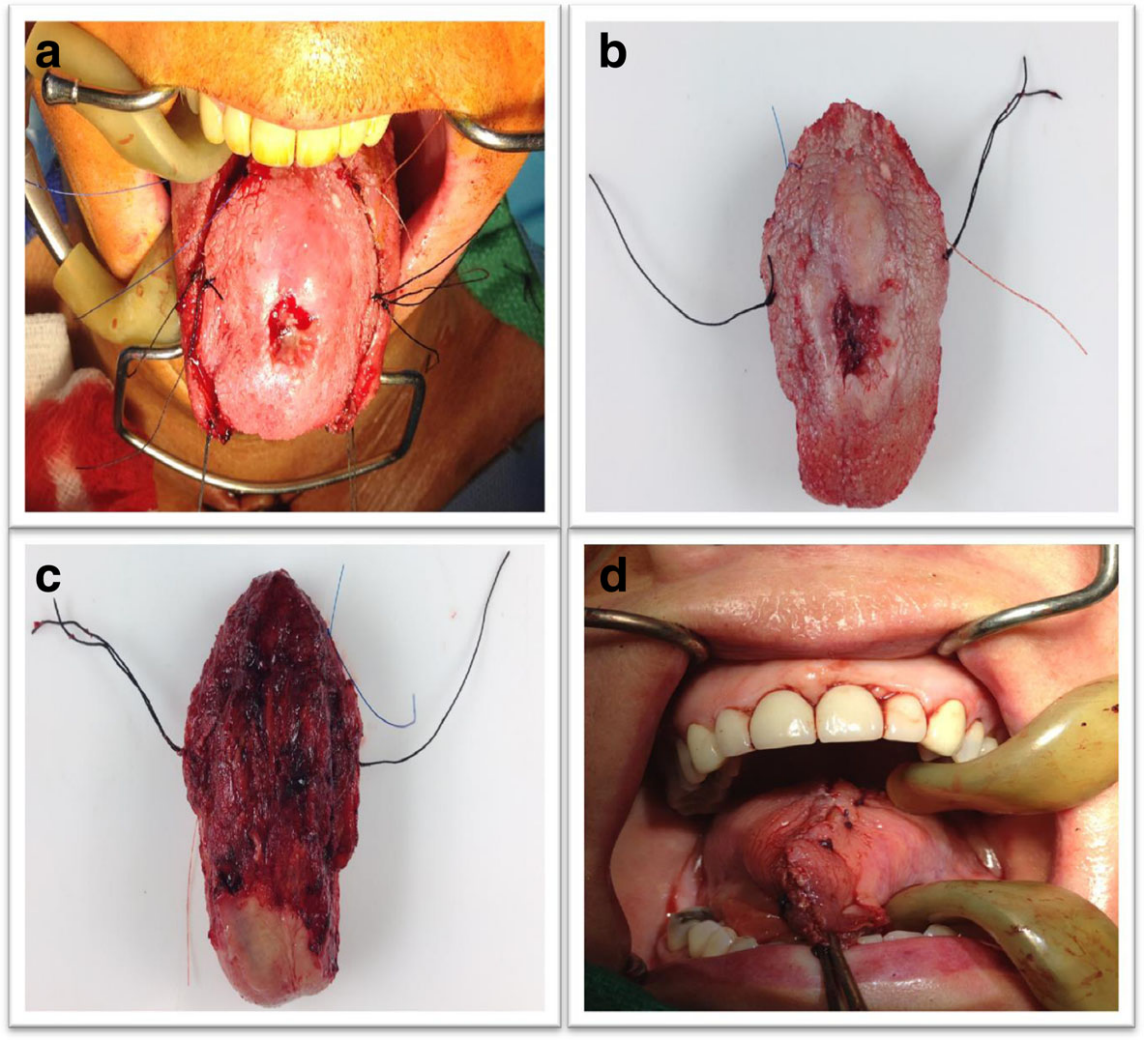
Surgical specimen from resection. Intraoperative Mohs margins. Image (**a**) demonstrates the surgical specimen about to be resected with proper orientation in both patient and the specimen. Images (**b** and **c**) demonstrate the resected specimen with corresponding sutures. Image (**d**) demonstrates closing time at the end of the operation once all margins were negative

The detailed surgical technique for total Mohs technique for oral cavity cancer resection has been previously described (Fig. 3) [7, 8]. Globally, the cancer is taken with adequate macroscopic margins (1 cm). Either sutures or ink are placed on this pathological specimen and on patient’s resection site. Sutures are placed in front of each other for further orientation (Fig. 3a). This also implies putting sutures in the deepest part of the resection to address the deep margins (Fig. 3b, c). Once in the pathological lab, the specimen is separated in quarters and oriented again. Each of these quarters is cut horizontally in thin layers in their entirety (Fig. 1). The pathologist assesses every slice and can precisely locate positive margins. The process starts again if margins are positive. When feasible, cancer resection is performed at the beginning of surgery and the specimen is sent for pathologic assessment during neck dissection.

### Protocol

The research protocol was submitted and approved by the research ethics committee of our institution before chart review was initiated. We retrospectively reviewed all patients who underwent oral cavity surgery for an oral cavity squamous cell carcinoma in an operative room (OR) setting. These surgeries occurred in 3 different hospitals: 2 tertiary care centers in head and neck surgery (Centre Hospitalier de l’Enfant-Jésus (HEJ), Quebec City, QC, Canada and Hôpital Notre-Dame (HND), Centre Hospitalier de l’Université de Montréal, Montreal, QC, Canada) and a community regional hospital (Centre Hospitalier de Lanaudière, St-Charles-Borromée (SCB), QC, Canada). The experimental group consisted of patients who underwent total Mohs technique for primary oral cavity cancer from August 2007 to August 2013 in those three centers (HEJ = 54, HND = 4, SCB = 2). Since total Mohs technique was adopted for almost all oral cavity squamous cell carcinoma since August 2007 in the first center mentioned above (HEJ), the control group consisted of patients operated with conventional surgery margins between August 2002 and August 2007 at only one of the two head and neck surgery centers (HEJ, *n* = 57).

Only patients presenting a squamous cell carcinoma of the oral cavity were included in the study, the hospital chart had to be complete and each patient had to have at least one year of follow-up. Patients were excluded if they had an oral cavity cancer within 5 years of the current diagnosis, another synchronous primary cancer, or prior radiation therapy for a head and neck cancer. Loco-regional recurrence was defined as a tissue-proven cancer recurrence at the primary site or in the regional lymph nodes within 5 years of the initial treatment. Time of recurrence was defined as the time between the date of the surgery and cancer recurrence on a pathology specimen.

### Analysis

Demographic data included patient’s age, sex, general and specific head and neck co-morbidities, complication rate, American Society of Anesthesiologists (ASA) physical status classification and hospitalization duration [11]. Surgical and pathological variables were also included.

Statistical analysis included univariate and bivariate analysis for demographic data. Two-sided Fisher exact test, Chi-square and Student *t*-test were used for that purpose. Z-test and Kaplan-Meier curves and log-rank test were performed to test the difference in the cancer recurrence between the Mohs and conventional methods. Univariate cox proportional hazard regression models were performed to select potential confounder variables. A *p*-value less than 0.10 was used for this preliminary step. Next, a multivariate cox proportional hazard regression models was performed to observe the difference in the cancer recurrence between the two methods in adjusting for potential confounder variables retained. Adjusted hazard ratios were reported with their 95% confidence intervals. Results with *p*-values *<* 0.05 are considered statistically significant. Statistical analyses were realized with SAS 9.3. The database is included as an addional supporting file (Additional file 1).

## Results

**Fig. 4:**
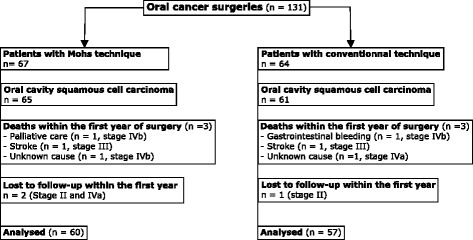
Flow chart of inclusion criteria

A total of 57 patients in the conventional group and 60 patients in the total Mohs margin group met the inclusion criteria. Figure 4 details patients excluded in each group. Demographic data are presented in Table 1. The mean age was similar in both groups (60.68 vs. 60.26 years, Student *t*-test, *p* = 0.859). There was a tendency for shorter feeding tube duration with total Mohs margin (10.89 days Mohs total margins vs. 14.46 days conventional surgery, Student *t*-test, *p* = 0.086). Flaps were performed in the same proportion for both groups (71.9% vs 60.0%, Chi-square, *p* = 0.174) without any difference in flap subtypes. The rest of the patients had primary and/or secondary closure. Using Mohs margins did not increase the length of the surgical procedure. Surgeries without flap reconstruction also have a similar duration (197.4 vs 167.3 min, Student *t*-test, *p* = 0.532) for either of these techniques.

**Table 1:** General patients demographic data

Characteristics	Conventional	Mohs	*p*-values
Number of patients	57	60	
Age (years)	60.26 (57.49; 63.03) 95% CI	60.68 (56.88; 64.49) 95% CI	0.859^3^
Male:Female	2.2:1	1.6:1	0.444
Comorbidities (%)			0.837^b^
None	24.6	19.2	
Metabolic	28.1	32.7	
Cardio-vascular	38.6	36.5	
Respiratory	3.5	7.7	
Miscellaneous	5.3	3.8	
Flap(s) performed (%)	71.9	60.0	0.174^a^
Subtypes			
Radial forearm	74.4	88.2	
Anterolateral thigh	2.3	2.9	
Pectoralis major	4.7	2.9	
Fibula osteocutaneous	11.6	2.9	
Other	2.3	2.9	
Complication rate (%)	50.9	42.2	0.488^a^
Types of complications (%)			0.761^b^
Metabolic	17.2	13.0	
Cardio-respiratory	17.2	26.1	
Infectious	24.1	13.0	
Flap problems	37.9	39.1	
Miscellaneous	3.4	8.7	
Neck dissection (%)	80.7	76.7	0.595^a^
Tracheotomy performed (%)	40.43	35.8	0.627^a^
Feeding tube (%)	73.0	67.3	0.567^a^
Feeding tube duration (days)	14.46 (10.45; 18.47) 95% CI	10.89 (9.24; 12.53) 95% CI	0.0862^c^
ASA Class (%)			0.714^a^
1	14.0	19.2	
2	56.1	55.8	
3	29.8	25.0	
Surgery duration for all patients (minutes)	475.1 (411.5; 538.8) 95% CI	380.1 (326.7; 433.5) 95% CI	0.025^c^
Surgery duration when no flap performed (minutes)	*n* = 16 patients 197.4 (105.65; 289.1) 95% CI	*n* = 21 patients 167.3 (113.2; 221.5) 95% CI	0.533^c^
Surgery duration for patients with flap	*n* = 40 patients 588.8 (537.1; 640.4) 95% CI	*n* = 31 patients 498.1 (455.9; 540.3) 95% CI	0.010^c^

^a^Chi-square

^b^Fisher ^c^Student *t* test

**Fig. 5:**
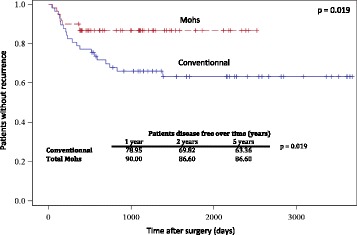
Kaplan Meier plot for cancer recurrence

Total Mohs margin technique demonstrated a lower loco-regional recurrence rate after one year of follow-up (10.0% vs 21.1%, Kaplan-Meier, *p* = 0.019). The recurrence rate still favors total Mohs margin at 5 years of follow-up (Fig. 5). Fig. 5 demonstrates the Kaplan Meier plot for cancer recurrence for unadjusted data. When considering only local tumor (Tis-T4N0M0), total Mohs margin technique significantly decreased loco-regional recurrence rate at 2 years when compared to conventional margin technique (10.5% vs 25.7%, z-score 1.849, *p* = 0.032). At 2 years of follow up, 6.6 patients with Tis-T4N0M0 squamous cell carcinoma of the oral cavity needed to receive total Mohs margin technique in order to prevent one recurrence (NNT 6.6). No conclusions after 2 years could be drawn due to a limited number of patients.

Table 2 shows the main pathological characteristics of the tumors. Cancer stage was similar in both groups. Deep margins were revised more often in total Mohs margin technique (48.6% of all margin vs 14.3, Chi-square, *p* = 0.086). More than 90% of patients with total Mohs margin technique achieved a clear margin of at least 2 mm compared with 83.6% of patients with conventional technique (Chi-square, *p* = 0.091). The mean closest margin was at 3.9 mm in conventional technique and 2.8 mm in Mohs total margin technique (Chi-square, *p* = 0.003). Mild dysplasia close to the margin was significantly less present in Mohs technique (15.4% vs 31.6%, Chi-square, *p* = 0.047). In cases of loco-regional recurrence, time to recurrence was not statistically different in conventional versus total Mohs technique (Chi-square, *p* = 0.082).

**Table 2:** Surgical and cancer characteristics

Characteristics	Conventional	Mohs	*p*-values
Cancer Stage (%)			0.216^a^
0	5.3	3.3	
I	19.3	25.0	
II	15.8	26.7	
III	31.6	15.0	
IV	28.1	30.0	
Infiltrative (%)	75.4	84.6	0.2332^b^
Number of margins taken after primary resection (%)			<0.0001^a^
0 margin	87.7	31.7	
1 margin	8.8	60.0	
2 margins	3.5	5.0	
3 margins	0	3.3	
Location of positive margins (%)			0.0861^b^
Floor of mouth	42.9	24.3	
Tongue base	28.65	5.4	
Deep margin	14.3	48.6	
Other	14.3	21.6	
Margins > 2 mm (%)	83.6	91.7	0.1883^b^
Closest margins dimension (mm)	3.949 (3.378; 4.520) 95% CI	2.785 (2.311; 3.258) 95% CI	0.0003^b^
Mild dysplasia close to margin (%)	31.6	15.4	0.0475^b^
Perineural or perivascular invasion (%)	21.1	29.6	0.2984^b^
Positive nodes dissection (%)	40.0	43.2	0.7547^b^
Zones for positive nodes (%)			0.8860^a^
I	20.0	15.4	
II	33.3	46.2	
III and further	46.7	38.5	
Capsular spreading (%)	15.8 (3/19)	35.3 (6/17)	0.2553^a^
Radiation therapy (%)	54.4	35.6	0.04191^b^
Radiation +/− Chemotherapy (%)	57.9	47.5	0.2604^b^
Time for cancer recurrence (median, days, IQR)	390.2 (234.8; 545.5)	182.5 (80.7; 284.3)	0.0832^b^
Follow-up time (median, days IQR)	2162 (1200, 2812)	1093 (532,4; 1444)	0.0100^b^
Cancer recurrence according to initial stages (% of recurrence)			
I	31.25	25	0.0913^a^
II	0	12.5	
III	67.50	25	
IV	31.25	37.5	

^a^Ficher exact test

^b^Chi-square test

Finally, multivariate analysis also confirms that poorly differentiated cancer (HR 3.676; 95% CI 1.330–10.164, *p* = 0.012), presence of ulceration (HR 2.725; 95% CI 1.211–6.130, *p* = 0.015) and perivascular invasion (HR 2.347; 95% CI 1.028–5.356, *p* = 0.0428) correlated with an increase in loco-regional recurrences. Main factors for adjustment included nodal stage, extracapsular spreading, margins status perineural/vascular/lymphatic invasion, sex, age, co-morbidities, past head & neck cancers. Statistically significant variants (*p* < 0.10) on univariate analysis included past head & neck cancers, neck dissection, histological grade, cancer.

## Discussion

### Loco-regional recurrence reduction

In our study, patients who underwent surgery with total Mohs margins technique had a statistically significant decrease in loco-regional recurrence rate when compared with conventional margin technique (Fig. 5). Demonstrating a decrease in local recurrence even for patients with N0 disease emphasizes the importance of negative margins. This is in agreement with Ganly et al. who demonstrated that positive surgical margins is the main independent predictor for local recurrence in patient with cT1T2N0 squamous cell carcinoma of the tongue [12].

In the total Mohs margins group; perioperative margin revision was more frequently required in order to obtain a definitive negative margin status. Almost 70% of patients in this group had another margin taken compared to only 12% in the conventional group. This could be explained by analyzing 100% of the tumor margins whereas conventional surgery only samples 0.1% of the margins. It seems only logical that positive margins are encountered more frequently with Mohs technique since 100% of the tumor is sampled. This necessarily requires additional margins to be taken at the time of surgery in order to achieve a negative final margin.

In our total Mohs margins technique group, positive margins are mainly situated at the deeper side (48.6%) of the tumor specimen. This is different from our conventional technique cohort, where positive margins are homogeneously distributed (Table 2). The increased frequency of positive margins at the deep part of the specimen might be specific to total Mohs margins technique. This has been previously suggested by Iseli et al. [13] who found no difference in the location of positive margins when retrospectively looking at their tumor specimens with conventional margins. This may be so because the removal of the deep part of the tumor is frequently done via palpation rather than visual and microscopic observation. This may lead to inaccurate deep margin location assessment in the tumor bed. The resection is initially done in a tridimensional fashion, which is altered by the resection itself. Once the tumor is removed, the surgical bed is modified and what was previously in three dimensions becomes flat and deformed, making tumor site identification more difficult. With total Mohs margins technique, the exact site of tumor persistence is easier to find since marking sutures are left in place on the patient side. It facilitates orientation for the surgeon and reproduction of tumor location from the pathologist map (Figs. 3 and 4). This is supported by findings from Kerawala et al. [14] who showed that the mean error in relocating the site of frozen section margin in oropharyngeal cancer is 9 mm for samples at the mucosal margin and 12 mm for samples placed deep into the tumor. Better tumor site identification may also explain the decrease in recurrence rates. Chang et al. [15] demonstrated a decrease in local recurrence rate when margins were taken directly from the pathologic specimen as opposed to margins from the tumor bed. As discussed by Davidson et al. [1], conventional frozen section analysis can evade microscopic tumor extensions. Therefore, it appears that total Mohs margins technique allows to counteract margin location variability by correctly geographically positioning the surgical margins as well as tracking tumor spread microscopically.

Our follow-up time is shorter for Mohs group compared to conventional group. However, all recurrences (except one patient in conventional group) occurred in the first three years of follow up. Thus, we are covering this more at-risk period well enough with our two groups.

Interestingly, more than 90% of patients with total Mohs technique had a clear margin of at least 2 mm compared to 83.6% in the conventional group. Achieving clear margins more often is a factor that contributes to the decrease of cancer recurrence locally. The mean distance of the closest margin seen on pathology is 3.9 mm in the conventional group versus 2.8 mm in the total Mohs margin technique group. This result may seem surprising. However, when considering that all margins are analyzed on multiple slices, we are confident that all these margins are truly negative in the total Mohs margin technique group [16]. Furthermore, the 2.8 mm distance of clear margin is very conservative as it represents the closest margin on the first specimen, not taking into account the margins sent again in the OR. Thus, we feel that 1–2 mm could easily be added to that number since it is generally the smallest amount of margin that can be additionally resected at one time. On the other hand, the 3.9 mm margin in the conventional group may have missed some positive margins that were not analyzed. Thus, the truly closest margin may be less than 3.9 mm since less than 1% of the margins are analyzed, compared to the 100% of margins with total Mohs margins technique (Figs. 1 and 4). Mohs margins were not possible to perform on bone. When mandibular bone was eroded, standard marginal or segmental mandibulectomy was performed to achieve local control of the disease. Mohs margins were performed for the remaining margins for those patients.

Definition of a close margin for carcinoma of the tongue does not have a standardized value. Close margins range from 2 to 5 mm in the literature, the latter being the most cited [17]. In the current study, 2 mm is defined as a negative margin. Total Mohs technique has previously been proven to be beneficial even with margin as small as 2 mm [16]. Ideally, a margin of 5 mm or more should be taken but many studies demonstrated no difference in outcomes between a 1–3 mm and 5 mm margin when the margin is truly negative [18]. Finally, a statistical tendency to mild dysplasia close to the surgical margin (<2 mm) is more frequent in the control group (31.6% vs 15.4%, Chi-square, *p* = 0.047). This finding may further contribute to the decrease in local recurrence in total Mohs margins group.

### Other benefits

When used judiciously, total Mohs margins did not increase operating time. In fact, reorganization in the OR setting led to a decrease in operating time when using Mohs technique as compared to conventional surgery. This reorganization involves resecting the tumor as the first step when feasible, followed by the neck dissection. Time from margins resection in the OR to pathology results to be available takes typically between 70 and 120 min. Neck dissection indications are mainly for advance tumor (T3-T4), positive or suspicious nodes prior to surgery and tumor thickness more than 4 mm. The decreased operating time may seem surprising, but both surgical and pathology teams gained experience with this technique over time. Furthermore, a second surgeon is now routinely involved in order to prepare the free flap while the first surgeon performs the oncological resection and neck dissection. This obviously contributes to a decreased surgical time independently of the total Mohs margin technique. However, if we consider only patients who did not require a free flap reconstruction during the surgery, thereby requiring only one surgeon, Mohs and conventional groups had similar surgery duration (197.4 min vs 167.3 min, Student *t*-test, *p* = 0.533). As for pathologists, their final pathology report is done at the end of the frozen margins report. Thus, this technique doesn’t increase their work time either. It simply concentrates their working time solely on the day of surgery for the total Mohs technique case. Pathologists with an interest or training with Mohs technique for skin lesion have the ability to transpose the technique to oral cavity cancer.

Mohs margins technique can be time consuming. Working in a tertiary care center with residents lengthens’ the duration of neck dissection giving more time to the pathologist to work. At the implantation of the technique, we had some down time in the OR to wait for revision margins. The key is the expertise gained by our pathologist’s team. We also informed them about the case days before the surgery, so they can allow enough resource to have a quick turnover for the margins.

Both groups required feeding tube in the same proportion after surgery. However, Mohs group had 3.4 fewer days with their feeding tube. Is tissue-sparing part of the explanation for this shorter length of enteral alimentation? Sparing more healthy tissue may contribute to the faster return of oral feeding due to lower rate of dysphagia. Lazarus et al. [19] reported that patients with tongue strength of at least 30 kPa performed better on functional and quality of life scales. The healthy tissue left in place may partially contribute to greater preservation of patients’ tongue strength and have a partial role in decreasing feeding tube time. Further assessments would be required in the future as historical bias could explainin part this difference.

### Limits of the study and future direction

This study has known limitations. First, it is - as previously mentioned - a retrospective study: no direct correlation could be assumed between total Mohs margin and a decrease in local recurrence. Furthermore, a decrease in recurrence does not imply a decrease in mortality, which is impossible to assess with current data. This study is also limited to patients with a new primary oral cavity cancer, thus data cannot be extrapolated to other population at this point. This study is also at risk of historical bias from our control group. However, both group had similar cancer stages and pathological characteristics. Furthermore, the same surgical team (limiting how surgical skills could impact the final results) performed the surgeries. We also had a limited number of patients.

Even though the number of cases is lower in the regional center, expertise or quality control was not an issue. This regional center actually developed and refined the technique. The Mohs’s technique is based on close communication between surgeons and pathologists. This strong link is essential in order to keep high standard of care at any center.

This technique can be challenging and requires a certain degree of expertise. The key point is having a pathological team ready to implant this technique, which can be time-consuming at the beginning. This technique also requires a team large enough to dedicate a pathologist and technician on the day of surgery. We understand that the current standard-of-care is the conventional margins that is widely performed in the vast majority of the centers.

Prospective data collection would be the next step of this study to support our current data. As in skin cancer, Mohs surgery is known for tissue sparing. It would be of interest to assess if this leads to a better oral function after surgery compared to conventional margin technique. Speech and swallowing function as well as a quality of life data would be interesting to analyze.

## Conclusions

Total Mohs margin seem associated with a decrease in loco-regional recurrence for oral cavity cancer compared to conventional margin technique, particularly for patients with N0 cancer. More importantly, this is not associated with an increase in operative time. Total Mohs margin technique analyses nearly 100% of the margins. It therefore required revising margins intra-operatively more often compared to conventional technique (including deep margins). Sharing knowledge between surgical and pathology teams greatly improved the expertise level for both teams. Therefore, our results strongly support the use of total Mohs margin technique to potentially decrease the loco-regional recurrence of oral cavity SCC tumors.

## Additional file

Additional file 1: Database. (XLSX 523 kb)

## Abbreviations

ASA: American Society of Anesthesiologists
HEJ: Hôpital Enfant-Jésus
HND: Hôpital Notre-Dame
NNT: Number needed to treat
OR: Operative room
SCB: Saint-Charles-Borroméee
SCC: Squamous cell carcinoma
